# Hemoadsorption Treatment with CytoSorb® in Probable Hemophagocytic Lymphohistiocytosis: A Role as Adjunctive Therapy?

**DOI:** 10.1155/2021/5539126

**Published:** 2021-08-20

**Authors:** Samuele Ceruti, Andrea Glotta, Harriet Adamson, Romano Mauri, Zsolt Molnar

**Affiliations:** ^1^Critical Care Department, Clinica Luganese Moncucco, Lugano, Switzerland; ^2^CytoSorbents Europe GmbH, Berlin, Germany; ^3^Centre for Translational Medicine, Semmelweis University, Budapest, Hungary; ^4^Department of Anaesthesiology and Intensive Therapy, Poznan University of Medical Sciences, Poznan, Poland; ^5^Department of Anaesthesiology and Intensive Therapy, Semmelweis University, Budapest, Hungary

## Abstract

Acute hemophagocytic lymphohistiocytosis (HLH) is a life-threatening disease, with an annual incidence of 1 : 800,000 people. The disease is characterized by a cytokine storm, with concomitant macrophage and natural killer (NK) cell activation; death can occur from multiple organ failure or complications such as bleeding diathesis. Therefore, HLH treatment remains a challenging one. We hereby present a case of a 76-year-old man with severe HLH in whom hemoadsorption was successfully applied. Due to the failure of the immunomodulatory therapy , continuous venovenous hemodiafiltration therapy with the CytoSorb® adsorber was successfully applied for 48 hours. Upon therapy discontinuation, the biological and clinical condition reverted, unfortunately evolving towards the patient's death.

## 1. Introduction

Acute hemophagocytic lymphohistiocytosis (HLH) is a highly fatal disease, with an incidence of 1 : 800,000 cases each year [1]. Reactive or secondary hemophagocytosis more frequently affects adults in response to an infectious stimulus, usually involving immune dysfunction [2]. The disease is characterized by a specific cytokine burst, in particular of the IFN-gamma, interleukin (IL) 6 and IL-18, and CD25 [3], associated with macrophage and NK-cell activation [4]. Death can occur due to tissue destruction, multiple organ failure, or complications such as bleeding diathesis [5]. Despite advances in critical care over the past decades, HLH treatment remains a challenging one. Hereby, we present a case of severe HLH in a complex scenario in whom hemoadsorption therapy was successfully used.

## 2. Case Report

### 2.1. Investigation

**Figure 1:**
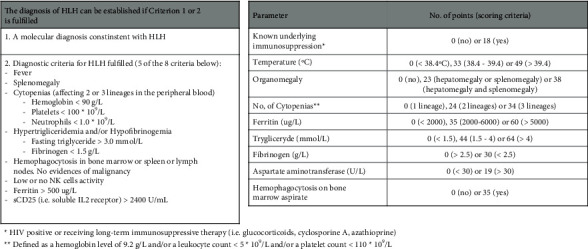
H-score calculator (H-score calculator redraw ([13])): the probability of HLH increases with the increase of score. The best cutoff value was 169, which corresponds to an accuracy of 90% (sensitivity of 93%, specificity of 86%). In this case report, patient presented a H-index of 229 points (96–99% of probability).

A 76-year-old man was admitted tothe Intensive Care Unit (ICU) for abdominal septic shock due to perforation of the colon; an obstructive colonic adenocarcinoma with metastatic lymph node spread was diagnosed immediately after the emergency laparotomy. Rapid resolution of the septic shock was managed after an emergency hemicolectomy followed by treatment with meropenem, vancomycin, and supportive treatment in ICU. However, on day 3, the patient developed refractory fever (around 40°C), altered level of consciousness (Glasgow Coma Scale 7/15), anemia (hemoglobin 6.8 g/dL), and thrombocytopenia (platelets 20 G/L) despite multiple replacements with blood products and hemodynamic support. Blood cultures remained negative during the entire ICU stay. Clinical course was further complicated by hemorrhagic shock for a massive right hemothorax (2,500 mL) as a result of a severe refractory thrombocytopenia, requiring fluid administration and high doses of norepinephrine (around 0.34 *μ*g/kg/min). Differential diagnosis was performed, which excluded other diseases. Heparin-induced thrombocytopenia [6] was excluded as a result of a low 4T-score pretest probability and the absence of anti-PF_4_ antibodies. Disseminated intravascular coagulation was also excluded due to relatively high fibrinogen levels and normal PT/aPTT according to the International Society of Thrombosis and Haemostasis (ISTH) score [7]. Thrombotic microangiopathy was excluded because of a negative schistocytes identification on smear evaluation at medium microscope magnification that was double checked [8]. Catastrophic antiphospholipid syndrome was also excluded as antiphospholipid antibodies were not identified [9]. High levels of serum ferritin (14,299 ng/mL), hypertriglyceridemia (4.49 mmol/L), and lactate dehydrogenase (LDH) elevation (1,354 U/L) supported the possibility of acute HLH. This was further reinforced by the H-score [10], which strongly suggested an acute HLH most likely triggered by septic shock due to the active cancer (Figure 1); bone marrow aspirate could not be obtained at this stage.

### 2.2. Diagnosis

Due to the high active infectious risk plus the diagnosis of metastatic colonic carcinoma, it was not feasible to proceed with the recommended immunomodulatory therapy with etoposide and corticosteroids, cyclosporin A, followed by bone marrow transplantation, according to the HLH-94 protocol [11]. Therefore, we started an alternative treatment of a 5-day cycle of immunoglobulin IV, which unfortunately failed to show any benefit. His clinical condition continued to deteriorate, with high norepinephrine doses, GCS around 6-7/15 points, and a continuous worsening of the inflammation and cell damage indices.

### 2.3. Treatment

Therefore, on day 7, an empiric hemoadsorption with the predialyzer CytoSorb® (Ultraflux AV 1000S filter) was started in combination with a neutral fluid balance [12]. A total of 48 hours of continuous hemoadsorption was performed, with each of the two adsorbers running for 24 hours.

### 2.4. Follow-Up and Outcome

**Figure 2:**
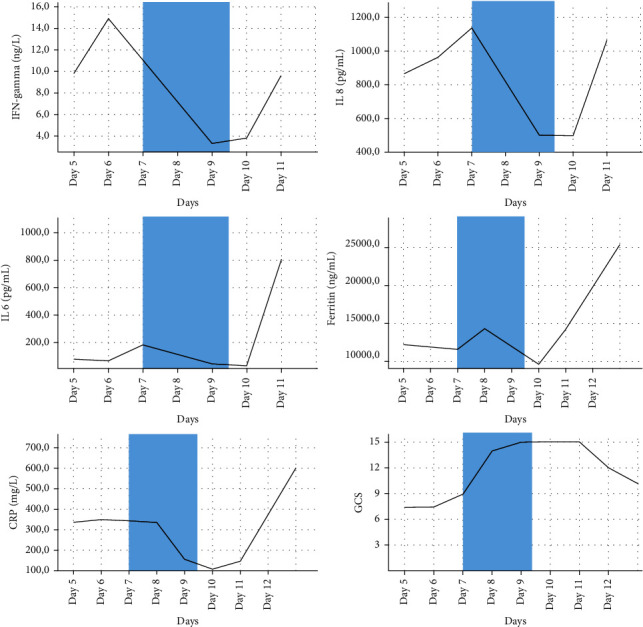
Temporal evolution of clinical and biological data: temporal evolution of IFN-gamma, interleukin (IL) 6 and IL8 cytokines' serum values, biological data like ferritin, C-reactive protein (CRP), and clinical data such as Glasgow Coma Scale (GCS). Days refer to the day of ICU treatment. Shaded blue areas are the days of CytoSorb® adsorbent application through CVVHDF.

From the start of the extracorporeal blood purification therapy, the patient's clinical and biochemical parameters improved, including creatinine (from 100 to 57 *μ*mol/L, Table 1); the patient regained consciousness (GCS 14/15) allowing endotracheal tube removal and complete norepinephrine cessation together with albumin replacement; inflammatory biomarker levels also improved (Table 1 and Figure 2), especially IFN*γ* (from 14.9 to 3.8 ng/mL, −74.5%), IL-8 (from 1137 to 497 pg/mL, −56.29%), IL-6 (from 183.4 to 30.6 pg/mL, −83.32%), ferritin (from 12209 to 9616 ng/mL, −21.24%), and C reactive protein (CRP from 343 to 108 mg/L, −68.51%). After 48 hours, the hemoadsorption treatment was stopped. However, on the following day, an unexpected deterioration occurred with worsening of both the clinical and laboratory values (up to +164% compared to the last day of hemoadsorption, Table 1 and Figure 2). The sudden worsening and the already compromised patient's clinical conditions prevented any further therapeutic approach with the hemoadsorption treatment. Despite all efforts, the patient died four days later.

## 3. Discussion

Acute HLH is a life-threatening disease, with an estimated incidence of one per 800,000 people [1]. Reactive or secondary HLH more frequently affects adults following an infectious stimulus in the context of certain predisposing factors and usually involves the dysregulated immune response [2]. The phenomenon is often characterized by cytokine storm, especially of IFN*γ*, IL-6, and IL-8 [3] with concomitant macrophage and NK-cell activation [4]. Death can occur due to tissue destruction, multiple organ failure, or complications such as bleeding diathesis [5]. In our case, the combination of septic shock, surgery inflicted tissue damage, and hemorrhagic shock led to an overwhelming inflammatory response, resulting in high ferritin levels and potentially fatal cytokine storm [13].

Sometimes, the recommended first-line therapy with etoposide and steroids may be contraindicated or associated with high risks, as is the case with a recent septic shock. Support with the CytoSorb® adsorber could be a valid treatment as a salvage therapy since it may remove both general inflammatory cytokines and the specific HLH cytokines (especially IL-8 and CD25). Clinical improvement and better survival rates could be the consequences [14].

In an HLH German registry concerning 137 patients, CytoSorb® has been applied as a rescue therapy in just 3 cases [14]. Although these data have been mainly demonstrated in the context of septic shock, some relevant trials have shown how hemoadsorption can be applied as an effective therapy compared to simple continuous venovenous hemodiafiltration (CVVHDF) for the removal of inflammatory cytokines and reducing high-dose catecholamine infusions, with quick reductions in procalcitonin (PCT) and C-reactive protein (CRP) levels [15,16]. The efficacy of CytoSorb® therapy in septic shock has not yet been proven in randomized controlled trials (RCT); the only two published RCTs demonstrated significant reductions in inflammatory cytokines in the treatment group, but this did not translate into an improvement in clinical outcomes [17,18]. Nevertheless, as compared to any type of dialysis or hemofiltration, hemoadsorption with CytoSorb® is fundamentally different: the cartridge contains biocompatible polymer beads, which allows a huge adsorption surface area as compared to dialysis filters, with an adsorption spectrum of up to 60 kDa [19].

Due to the rarity of HLH, data are scarce. As cytokines certainly play a pathogenic role in HLH [3,4], they could play a pivotal role in the observed high mortality rate [5]; hence, it has sound pathophysiological rationale to use hemoadsorption early in these patients. So far, studies concerning the effect of cytokine removal on mortality reduction are missing in general and certainly for such a rare disease as HLH. Therefore, our knowledge is limited to a few single case studies only [20,21]. Despite the negative eventual outcome, the current case report is one the first ones to show that at least temporary improvement of both clinical and biochemical parameters could be achieved within a matter of a couple of days. These data could be hypothesis generating for prospective studies in the future.

These data have been mainly demonstrated in the context of septic shock; some relevant trials have shown how hemoadsorption can be applied as an effective therapy compared to simple CVVHDF for the removal of inflammatory cytokines and reducing high-dose catecholamine infusions, with quick reductions in PCT and CRP levels [15,16]. CytoSorb® has been reported to be applied as a rescue therapy in 3 cases of HLH [14]. HLH in the context of sepsis presents a unique challenge as the treatment of HLH requires strong immunomodulation, which can potentially worsen sepsis. Our case report provides further support to the pathophysiological rationale that CytoSorb® could mitigate the cytokine storm with minimum immunomodulation; hence, it could provide an alternative therapeutic approach in this patient population.

## Figures and Tables

**Table 1:** Laboratory characteristics.

	Normal values	Day 5	Day 6	Day 7	Day 8	Day 9	Day 10	Day 11	Day 12	Day 13
Hematology										
Hemoglobin	14.0–18.0 (g/dL)	9.2	9.3	9.5	10.0	8.2	7.5	8.0		7.2
Red cells	4.5–6.0 (G/L)	3.19	3.23	3.26	3.42	2.77	2.52	2.69		2.38
Leucocyte	4.0–10.0 (G/L)	3.4	4.0	4.6	7.2	4.2	3.6	2.3		3.4
Thrombocyte	150–450 (G/L)	53.0	44.0	49.0	49.0	34.0	33.0	26.0		30.0

Coagulation										
Quick	70–130 (%)	110.0	110.0	94.0	70.0					63.0
INR		0.98	0.98	1.04	1.16					1.22
aPTT	26–36 (sec)	32.0	34.0	35.0	33.0					42.0
Fibrinogen	1.7–4.1(g/L)	5.34	5.07	4.66	4.22					5.57
D-dimer	<500 (ng/mL)		>9000	>9000	>9000					>9000

Chemistry										
Creatinine	62–105 (*μ*mol/L)	92.0	100.0	98.0	69.0	60.0	57.0	95.0		119.0
Albumin	35–52 (g/L)		19.0	22.0	19.0	27.0	32.0			22.0
Total bilirubin	<21 (*μ*mol/L)	7.5	7.0	9.6	8.6	13.5	14.6	18.4		10.1
Ferritin	30–50 (ng/mL)	12209		11586	14299	9616		14231		25408
LDH	135–225 (U/L)		1159.0	1461.0	1354.0	1177.0	1292.0	1898.0		2099.0
CK	39–308 (U/L)		221.0	242.0	243.0	234.0	239.0	307.0		349.0
GOT	10–50 (U/L)		133.0	152.0	119.0	136.0	131.0	240.0		253.0
GPT	10–50 (U/L)		51.0	43.0	35.0	33.0	26.0	41.0		59.0
Lipase	13–60 (U/L)				77.0	85.0	356.0	183.0		28.0
Calcium	2.13–2.65 (nmol/L)				2.56	2.44	2.45			2.28
Phosphate	0.81–1.45 (nmol/L)				1.54	1.58	1.48	1.88		2.59
CRP	<5 (mg/L)	349	343	335	155	108	146			601
Procalcitonin	<0.05 (*μ*mol/L)	1.21	1.16	1.49	1.74	1.56	1.18	1.3		15.1

Immunology										
IFN*γ*	<15 (ng/L)	9.8	14.9	11.2	3.3	3.8	9.6			
IL8	<31.2 (pg/mL)	963	1137		499	497	1064			
IL6	<6.4 (pg/mL)	79.5	67.4	183.4	45.1	30.6	807			

Laboratory characteristics. Days 7–9 correspond to CytoSorb® adsorbent application through CVVHDF.

## Data Availability

The data used to support the findings of this study are available from the corresponding author upon request.
